# Olive allergens under the microscope: Ole e 7 and Ole e 9 sensitization does not increase symptom severity or adverse reactions to subcutaneous allergen immunotherapy^☆^

**DOI:** 10.1016/j.waojou.2026.101482

**Published:** 2026-11-04

**Authors:** Manuel Alcántara Villar, Alicia López Guerrero, Miriam Bermúdez Bejarano, Cristina Ruiz González, Patricia de Manueles Tabliega, Guillermo Salamanca Fernández

**Affiliations:** a Allergology Unit, Jaén University Hospital, Jaén, Spain; b Medical Affairs Unit, LETI Pharma, Madrid, Spain

**Keywords:** Olive pollen, Allergic respiratory disease, Component-resolved diagnosis, Allergen immunotherapy, Subcutaneous immunotherapy, Safety

## Abstract

**Background:**

In regions with prolonged and intense exposure to olive pollen, sensitization to minor allergens such as Ole e 7 and Ole e 9 and their specific IgE levels tend to increase. This has raised concerns regarding a potentially higher risk of adverse reactions during allergen immunotherapy.

**Objective:**

To assess subcutaneous immunotherapy (SCIT) safety and the clinical outcomes observed after 1 year of treatment in routine clinical practice in patients with olive pollen–induced allergic respiratory disease, according to their molecular sensitization profiles.

**Methods:**

This retrospective observational study included patients with rhinitis and/or asthma caused by olive pollen sensitization who received SCIT with a depigmented, polymerized olive pollen extract. Olive allergen sensitization profiles were determined using component-resolved diagnosis, and their association with disease severity, treatment tolerance, and clinical response was assessed.

**Results:**

A total of 196 patients were included in the analysis. Rhinitis was present in 97% of patients and asthma in 94%, predominantly of moderate persistent severity. Sensitization to Ole e 1 was observed in 96% of patients, whereas 73% and 68% were sensitized to Ole e 7 and Ole e 9, respectively; 58% were sensitized to all 3 components. Six late local reactions and 1 grade 3 systemic reaction (EAACI classification) were reported. Clinical improvement according to the predefined clinical response criteria was observed in 76% of patients, along with a reduction in rescue medication use and a decrease in the number of patients requiring emergency department visits, from 60 at baseline to none after 1 year of follow-up. No association was observed between molecular sensitization profiles and SCIT tolerability or clinical response.

**Conclusion:**

SCIT with a depigmented polymerized olive pollen extract was well tolerated and was associated with favorable one-year clinical outcomes in a population with high environmental exposure to olive pollen, irrespective of sensitization to minor olive allergens.

## Introduction

Allergic respiratory diseases (ARD), including rhinitis and asthma, are triggered by exposure to allergens such as pollen, dust mites, and animal dander, and have a major impact on quality of life.^1^^,^^2^ Pollen is a leading cause of ARD worldwide, and its prevalence is increasing due to air pollution and climate change.^3^^,^^4^ Olive pollen (*Olea europaea*) is the main cause of respiratory allergy in the Mediterranean, which hosts 95% of the world's olive trees,^5^ though it is also relevant in regions of the Americas, South Africa, Japan, and Australia.^6^ In Spain, olive pollen ranks second after grasses and is the leading cause of ARD in southern areas such as Jaén and Córdoba, where daily peaks can exceed 5000 grains/m^3^, often triggering severe symptoms and asthma exacerbations.^7^, ^8^, ^9^, ^10^

Olive pollen has a complex allergenic profile, with 15 recognized allergens (Ole e 1 to Ole e 15)^11^ Ole e 1 is the major allergen, detected in up to 80% of sensitized individuals,^12^ while polysensitization —common in high-exposure regions— is a recognized risk factor for ARD and is associated with increased disease severity.^13^, ^14^, ^15^ In areas with prolonged and extremely high pollen counts, such as Jaén, minor allergens like Ole e 7 and Ole e 9 become clinically relevant.^16^^,^^17^ Some studies suggest that Ole e 7 may act independently of Ole e 1, with up to 5% of patients showing monosensitization to this allergen.^18^, ^19^, ^20^ This complexity challenges conventional diagnosis, making component-resolved diagnosis (CRD) essential for accurate IgE profiling.^21^

Allergen immunotherapy (AIT) is the only disease-modifying treatment for IgE-mediated allergies, though its efficacy and safety can vary among patients and according to sensitization profile.^22^ Sensitization to Ole e 7 and Ole e 9 has been suggested to be associated with reduced AIT tolerance and higher risk of asthma,^17^, ^18^, ^23^, ^24^, ^25^ Current guidelines generally do not recommend AIT for patients who are not sensitized to the major allergen of a given source, unless products with controlled minor allergen doses are available.^26^^,^^27^ CRD allows AIT to be tailored to each patient's specific sensitization profile, potentially improving outcomes.^21^^,^^28^

This study assessed the safety of subcutaneous immunotherapy (SCIT) and the clinical outcomes observed after 1 year of treatment with a depigmented polymerized olive pollen extract in routine clinical practice among patients with olive pollen-induced ARD in Jaén, Spain. Clinical outcomes were evaluated according to patients' sensitization profiles to Ole e 1, Ole e 7, and Ole e 9 in this region of high olive pollen exposure.

## Methods

### Study design and population

This retrospective observational study analyzed real-world data from pediatric and adult patients (5–55 years) with seasonal ARD (clinically diagnosed rhinitis and/or asthma) due to olive pollen sensitization. Patients treated at Jaén University Hospital were retrospectively identified and their medical records were subsequently reviewed between February and March 2020. Eligible patients met the following criteria: (i) a positive skin prick test (mean wheal diameter ≥3 mm) and/or serum specific IgE >0.70 kU/L (CAP class ≥2) to whole olive pollen extract, according to the routine diagnostic criteria used in our department during the study period; (ii) at least 2 years of disease evolution; (iii) sIgE determination for Ole e 1, Ole e 7, and Ole e 9; and (iv) treatment with SCIT using a depigmented polymerized *O. europaea* pollen allergen extract (Depigoid; LETI Pharma, S.L.U.) between January 2014 and December 2019. Skin prick testing and serum specific IgE determinations were performed as part of routine clinical care before study inclusion and not specifically for research purposes. The only exclusion criterion was an incomplete medical record that precluded evaluation of the predefined study variables, including molecular sensitization profile, immunotherapy tolerance, and clinical response. Of 196 potentially eligible medical records identified, none were excluded because of incomplete data, and all 196 patients were included in the final analysis. The decision to prescribe AIT, including the assessment of indications and contraindications, had been made by the treating allergist before study inclusion in accordance with routine clinical practice and the applicable clinical guidelines during the study period. All data were anonymized before analysis.

### Objectives and variables

The primary objective was to describe olive pollen sensitization profiles in patients with seasonal ARD from Jaén (Spain) using component-resolved diagnosis (CRD) and to assess the safety of SCIT with a depigmented polymerized *O. europaea* pollen allergen extract according to molecular sensitization profile.

Secondary objectives included evaluating the association between molecular sensitization and the type and severity of ARD (rhinitis and asthma), as well as clinical response at 1 year.

Specific IgE to Ole e 1, Ole e 7, and Ole e 9 was measured using ImmunoCAP (Thermo Fisher Scientific). According to the manufacturer's recommendations, values ≥ 0.35 kU/L were considered indicative of sensitization to each molecular component. ARD severity was classified using ARIA guidelines for rhinitis^29^ and GEMA v4.2 guidelines for asthma.^30^ Additional variables included demographics (age, sex), clinical variables (diagnosis, family history, comorbid allergies, disease duration), and immunotherapy outcomes.

(i) Treatment tolerance: Occurrence or absence of adverse reactions (AR).
(ii) Clinical response: Clinical responders were defined as patients who reported symptomatic improvement confirmed by the treating physician and supported by improvement in at least 1 documented clinical or healthcare-utilization outcome between baseline and the one-year follow-up: reduced use of rescue or maintenance medication, reduced or discontinued systemic corticosteroid use, or fewer emergency department visits. Adverse reactions were assessed separately as safety and tolerability outcomes and were not considered in the assessment of clinical response.
(iii) Owing to the retrospective design of the study, standardized pulmonary function measures, including spirometry and peak expiratory flow (PEF), were not systematically available at predefined baseline and follow-up time points and were therefore not included in the evaluation of clinical response.

### Statistical analyses

Qualitative variables were summarized as frequencies and percentages; quantitative variables as mean (SD) or median (Q1; Q3). Asthma severity was grouped into 4 categories (intermittent, mild persistent, moderate persistent, severe persistent) and rhinitis into 3 categories (mild intermittent, mild persistent, moderate–severe persistent) for analysis. Associations between sensitization profile, tolerance to AIT, and clinical response were evaluated. Statistical significance was set at two-sided α < 0.05. Analyses were performed using GNU PSPP® v1.2.0. Variance homogeneity was assessed with Levene's test; parametric tests (Student's t, ANOVA) or non-parametric tests (Mann–Whitney U, Kruskal–Wallis) were applied as appropriate. Categorical variables were compared using chi-square tests.

### Ethical considerations

Due to the observational nature of the study, patient consent was waived by the Research Ethics Committee. The study complied with GDPR (EU 2016/679), Spain's Organic Law on Data Protection (December 2018), the Helsinki Declaration, Good Clinical Practice, and current Spanish regulations. Approval was obtained from the Research Ethics Committee of the province of Jaén.

## Results

### Study population

A total of 196 potentially eligible medical records were retrospectively identified. No records were excluded because of incomplete data, and all 196 patients were included in the final analysis. All patients had olive pollen allergy and had received SCIT with a depigmented polymerized olive pollen allergen extract. The study population comprised 106 males (54.1%) and 90 females (45.9%), with a median (Q1; Q3) age of 17 (10; 27) years (14 years in males and 20 in females). All had a positive skin prick test to olive pollen. Among the 100 patients with available measurements, mean (SD) serum specific IgE to olive pollen was 56.5 (40.3) kU/L.

Rhinitis was present in 97.4% (191/196) of patients and was classified as moderate-to-severe persistent in 86.9% (166/191). Asthma was present in 94.4% (185/196), predominantly of moderate persistent severity (72.4% [134/185]), whereas only 1.6% (3/185) had severe persistent asthma. Rhinitis and asthma coexisted in 91.8% (180/196) of patients. A family history of atopy was present in 55.6% (109/196), while 12.8% (25/196) had atopic dermatitis and 20.9% (41/196) had food allergy. Mean (SD) disease duration was 6.5 (5.7) years, with similar values observed in males and females (6.0 and 7.1 years, respectively) (Table 1).

**Table 1:** Baseline demographic and clinical characteristics of patients.

Characteristic	N = 196
**Sex**, n (%)	
Male	106 (54.1)
Female	90 (45.9)
**Age**, median (Q1; Q3) – years	
All	17 (10; 27)
Males	14 (10; 23)
Females	20 (12; 29)
**Clinical characteristics**, n (%)	
Family history of atopy	109 (55.6)
Atopic dermatitis	25 (12.8)
Positive SPT with olive pollen allergen extract	196 (100.0)
Total IgE – kU/mL	
Mean (SD)	713.4 (828.5)
Median (Q1; Q3)	358.0 (174.0; 938.0)
sIgE to olive tree pollen, mean (SD) – kU/mL	
Mean (SD)	56.5 (40.3)
Median (Q1; Q3)	59.5 (11.8; 100.0)
Food allergy	41 (20.9)
Eggs	15 (7.7)
Milk	4 (2.0)
Oral Allergy Syndrome	12 (6.1)
Rhinitis	191 (97.4)
Rhinitis (ARIA scale)	
Mild – intermittent	2 (1.0)
Mild – persistent	23 (11.7)
Moderate – severe persistent	166 (84.7)
Asthma	185 (94.4)
Asthma severity (GEMA 4.2)	
No	11 (5.6)
Intermittent	22 (11.2)
Mild – persistent	27 (13.8)
Moderate – persistent	134 (68.4)
Severe – persistent	3 (1.5)
Rhinitis + asthma	180 (91.8)
**Years of disease evolution**, mean (SD) – years	
All	6.5 (5.7)
Males	6.0 (5.4)
Females	7.1 (6.1)
**Allergen immunotherapy**	
Type of vaccine, n (%)	
Depifast	58 (29.6)
Depigoid	72 (36.7)
Depigoid duo	47 (24.0)
Depigoid 3	19 (9.7)
Vaccine composition, n (%)	
Monosensitized	78 (39.8)
Polysensitized (mix)	118 (60.2)

Abbreviations: ARIA, Allergic Rhinitis and its Impact on Asthma guidelines; GINA, Global Initiative for Asthma guidelines; Q1, first quartile; Q3, third quartile; SD, standard deviation; SPT, skin prick test.

### Molecular sensitization profile

Sensitization to Ole e 1 was the most prevalent, detected in 95.9% of patients (mean [SD] sIgE: 46.4 [40.1] kU/L), followed by Ole e 7 in 73.0% (35.7 [38.3] kU/L) and Ole e 9 in 67.9% (38.7 [34.6] kU/L). Most patients were polysensitized, 57.7% to all 3 allergens and 23.2% to 2 allergens, whereas 15.8% were monosensitized to Ole e 1 and 2.0% to Ole e 7 only. Triple-negative patients were rare (1.5%), as were those sensitized only to Ole e 7 and/or Ole e 9 without Ole e 1 (2.5%) (Table 2).

**Table 2:** Allergen sensitization profiles.

Single sensitization profiles	
**Olive complete extract positives**	
All positives, n (%)	100 (100.0)^a^
sIgE, mean (SD) – kU/L	56.5 (40.3)
Males, n (%)	59 (59.0)
sIgE, mean (SD) – kU/L	59.9 (39.4)
Females, n (%)	41 (41.0)
sIgE, mean (SD) – kU/L	51.5 (41.6)
**Ole e 1 positives**	
All positives, n (%)	188 (95.9)
sIgE, mean (SD) – kU/L	46.4 (40.1)
Males, n (%)	103 (54.8)
sIgE, mean (SD) – kU/L	53.4 (40.5)
Females, n (%)	85 (45.2)
sIgE, mean (SD) – kU/L	37.8 (38.1)
**Ole e 7 positives**	
All positives, n (%)	143 (73.0)
sIgE, mean (SD) – kU/L	35.7 (38.3)
Males, n (%)	83 (58.0)
sIgE, mean (SD) – kU/L	31.3 (36.8)
Females, n (%)	60 (42.0)
sIgE, mean (SD) – kU/L	41.7 (39.8)
**Ole e 9 positives**	
All positives, n (%)	133 (67.9)
sIgE, mean (SD) – kU/L	38.7 (34.6)
Males, n (%)	75 (56.4)
sIgE, mean (SD) – kU/L	39.4 (36.3)
Females, n (%)	58 (43.6)
sIgE, mean (SD) – kU/L	37.7 (32.4)
**Multiple sensitization profiles, n (%)**	
Ole e 1^+^/Ole e 7^+^/Ole e 9^+^	113 (57.7)
Males^b^	66 (58.4)
Females	47 (41.6)
Ole e 1^+^/Ole e 7^+^/Ole e 9^−^	25 (12.8)
Males	15 (60.0)
Females	10 (40.0)
Ole e 1^+^/Ole e 7^−^/Ole e 9^+^	19 (9.7)
Males	9 (47.4)
Females	10 (52.6)
Ole e 1^+^/Ole e 7^−^/Ole e 9^−^	31 (15.8)
Males	13 (41.9)
Females	18 (58.1)
Ole e 1^−^/Ole e 7^+^/Ole e 9^+^	1 (0.5)
Males	0 (0.0)
Females	1 (100.0)
Ole e 1^−^/Ole e 7^+^/Ole e 9^−^	4 (2.0)
Males	2 (50.0)
Females	2 (50.0)
Ole e 1^−^/Ole e 7^−^/Ole e 9^−^	3 (1.5)
Males	1 (33.3)
Females	2 (66.6)

Abbreviations: SD, standard deviation; sIgE, specific immunoglobulin E.

a Serum sIgE levels to olive tree pollen were determined in 100 patients, all of whom were positive.

b The percentages of males and females were calculated considering the total number of positives in each allergen sensitization profile as 100%.

### Tolerance to immunotherapy

Most patients tolerated AIT regardless of their sensitization profiles. For Ole e 1, tolerance was 96.3% (181/188) in sensitized patients and 100% (8/8) in non-sensitized. For Ole e 7, rates were 95.8% (137/143) vs. 98.1% (52/53), and for Ole e 9, 96.2% (128/133) vs. 96.8% (61/63), respectively (Fig. 1A). For multiple sensitization profiles, the percentage of tolerant patients was 96.5% (109/113) for Ole e 1^+^/Ole e 7^+^/Ole e 9^+^ profile, 94.7% (18/19) for Ole e 1^+^/Ole e 7^-^/Ole e 9^+^, 92.0% (23/25) for Ole e 1^+^/Ole e 7^+^/Ole e 9^-^, and 100.0% (31/31) for Ole e 1^+^/Ole e 7^-^/Ole e 9^-^. The number of patients in each of the 3 remaining sensitization profiles analyzed —all negative for Ole e 1—was very low, and therefore results should be taken with caution: 100.0% (1/1) for Ole e 1^-^/Ole e 7^+^/Ole e 9^+^, 100.0% (4/4) for Ole e 1^-^/Ole e 7^+^/Ole e 9^-^, and 100.0% (3/3) for Ole e 1^-^/Ole e 7^-^/Ole e 9^-^ (Fig. 1B).

**Fig. 1:**
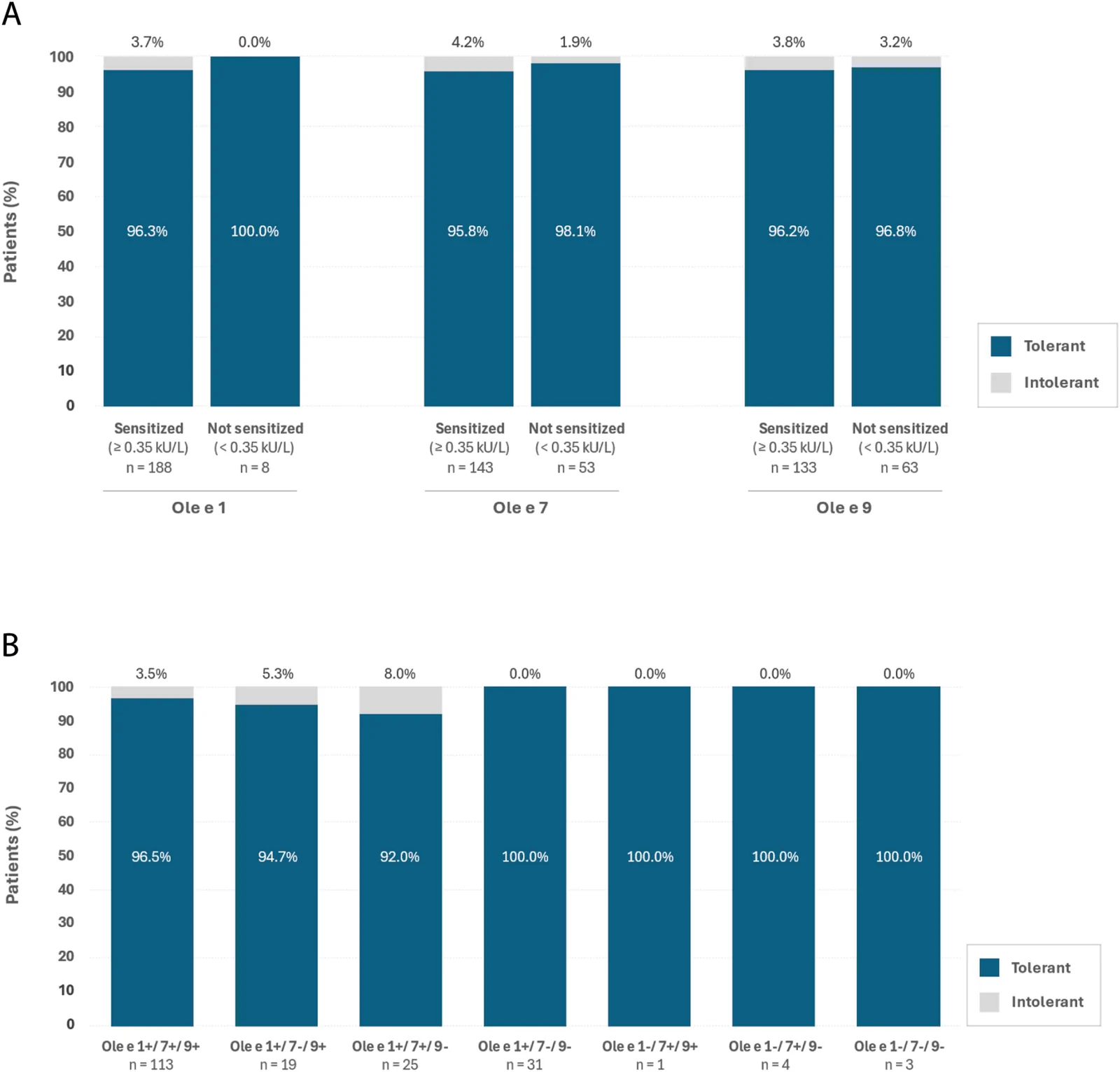
**Tolerance to allergen immunotherapy according to sensitization profile**. Percentage of patients exhibiting tolerance (dark shading) or intolerance (light shading) to allergen immunotherapy, stratified by their sensitization to olive pollen allergens. **(A)** Patients categorized according to sensitization to individual allergens (Ole e 1, Ole e 7, Ole e 9). **(B)** Patients categorized according to sensitization to multiple allergens, as indicated

### Adverse reactions

Seven treatment-related adverse reactions were reported, including 6 delayed local reactions (85.7%) and 1 delayed systemic reaction (14.3%). Local reactions consisted of erythema, edema, pruritus, pain, and warmth at the injection site; 4 were classified as mild and 2 as moderate. The only systemic reaction (grade 3 according to the EAACI classification) occurred during the build-up phase in a six-year-old boy with a three-year history of well-controlled moderate persistent asthma, moderate-to-severe persistent allergic rhinitis, atopic dermatitis, and a family history of olive pollen allergy. The patient was sensitized to Ole e 1 and Ole e 7, but not to Ole e 9, and was receiving a mixture of depigmented polymerized allergen extracts containing 70% olive pollen and 30% grass pollen. The reaction occurred after the first dose, administered outside the olive pollen season, and consisted of cough and dyspnea accompanied by a moderate local reaction at the injection site. Symptoms resolved promptly following treatment with inhaled salbutamol and systemic corticosteroids, without the need for hospitalization or discontinuation of immunotherapy. The patient subsequently completed the treatment course without experiencing any further local or systemic adverse reactions. No serious adverse reactions were observed during the study (Table 3). By sensitization profile, it is worth noting that no AR were reported in Ole e 1-negative patients (Fig. 2).

**Table 3:** Treatment-related adverse reactions.

Patient #	Sex	Age^a^	Type	Signs/symptoms	Intensity^b^	Onset^b^	Severity	Allergen profile
217	Female	20	LR	Erythema, edema, pruritus, pain and warmth	Mild	Delayed	No	Ole e 1+/Ole e 7−/Ole e 9+
254	Male	23	LR	Erythema, edema, pruritus, pain and warmth	Mild	Delayed	No	Ole e 1+/Ole e 7+/Ole e 9+
430	Male	22	LR	Erythema, edema, pruritus, pain and warmth	Moderate	Delayed	No	Ole e 1+/Ole e 7+/Ole e 9−
639	Male	8	LR	Erythema, edema, pruritus, pain and warmth	Moderate	Delayed	No	Ole e 1+/Ole e 7+/Ole e 9+
811	Female	20	LR	Erythema, edema, pruritus, pain and warmth	Mild	Delayed	No	Ole e 1+/Ole e 7+/Ole e 9+
855	Female	29	LR	Erythema, edema, pruritus, pain and warmth	Mild	Delayed	No	Ole e 1+/Ole e 7+/Ole e 9+
987	Male	6	SR^c^	Erythema, cough and dyspnea	Grade 3	Delayed	No	Ole e 1+/Ole e 7+/Ole e 9−

Abbreviations: LR, local reaction; SR, systemic reaction.

a Age was expressed in years.

b Systemic reactions classified according to the grading system of the European Academy of Allergy and Clinical Immunology (EAACI).

c The reaction occurred after the first dose, administered outside the olive pollen season. Symptoms resolved promptly following treatment with inhaled salbutamol and systemic corticosteroids, without the need for hospitalization or discontinuation of immunotherapy. The patient subsequently completed the treatment course without experiencing any further local or systemic adverse reactions.

**Fig. 2:**
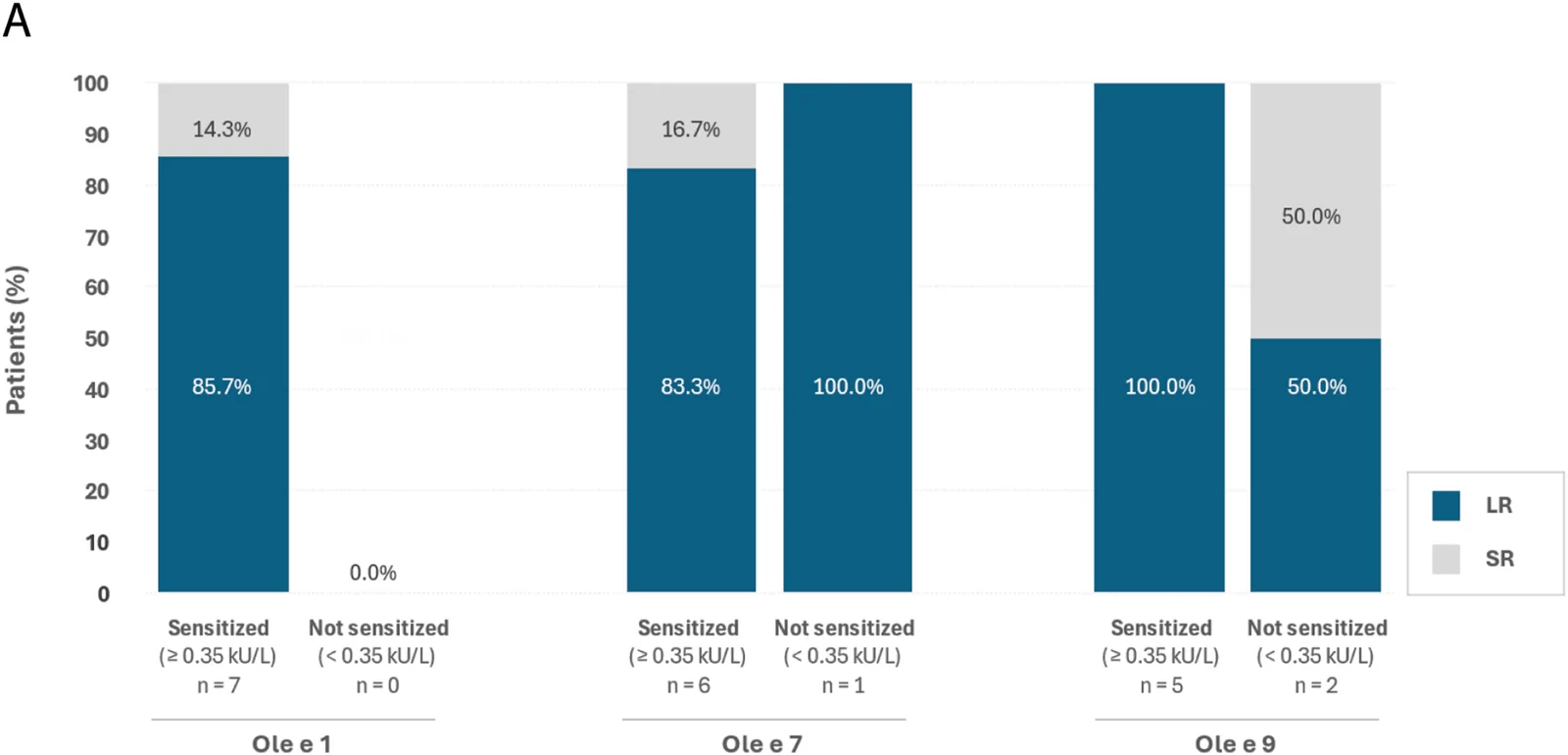
**Adverse reactions to allergen immunotherapy according to sensitization profile**. Percentage of patients experiencing local (LR, dark shading) or systemic (SR, light shading) adverse reactions to allergen immunotherapy, stratified by sensitization to individual olive pollen allergens (Ole e 1, Ole e 7, Ole e 9)

### Clinical response at one-year follow-up

At 1 year, 149 of 196 patients (76.0%) met the predefined criteria for clinical response. The proportion of patients meeting these criteria was similar among those sensitized to Ole e 1 (77.7%; 146/188), Ole e 7 (75.5%; 108/143), and Ole e 9 (76.7%; 102/133). Comparable proportions were observed among patients non-sensitized to Ole e 7 (77.4%; 41/53) or Ole e 9 (74.6%; 47/63). In contrast, only 37.5% (3/8) of Ole e 1–negative patients met the predefined clinical response criteria. An association was observed between Ole e 1 sensitization and the likelihood of meeting the predefined clinical response criteria at 1 year (*p* = 0.009); however, this finding should be interpreted with caution, because only 8 patients were negative for Ole e 1 (Fig. 3A). Across multiple sensitization profiles, the majority of patients with the Ole e 1^+^/Ole e 7^+^/Ole e 9^+^, Ole e 1^+^/Ole e 7−/Ole e 9^+^, Ole e 1^+^/Ole e 7^+^/Ole e 9−, Ole e 1^+^/Ole e 7−/Ole e 9−, and Ole e 1−/Ole e 7^+^/Ole e 9^+^ profiles met the predefined clinical response criteria, with proportions ranging from 68.4% to 100.0%. Conversely, most patients with the Ole e 1−/Ole e 7+/Ole e 9− and Ole e 1−/Ole e 7−/Ole e 9− profiles did not meet these criteria. As the number of Ole e 1− patients was very limited, findings for these subgroups should be interpreted with caution (Fig. 3B).

**Fig. 3:**
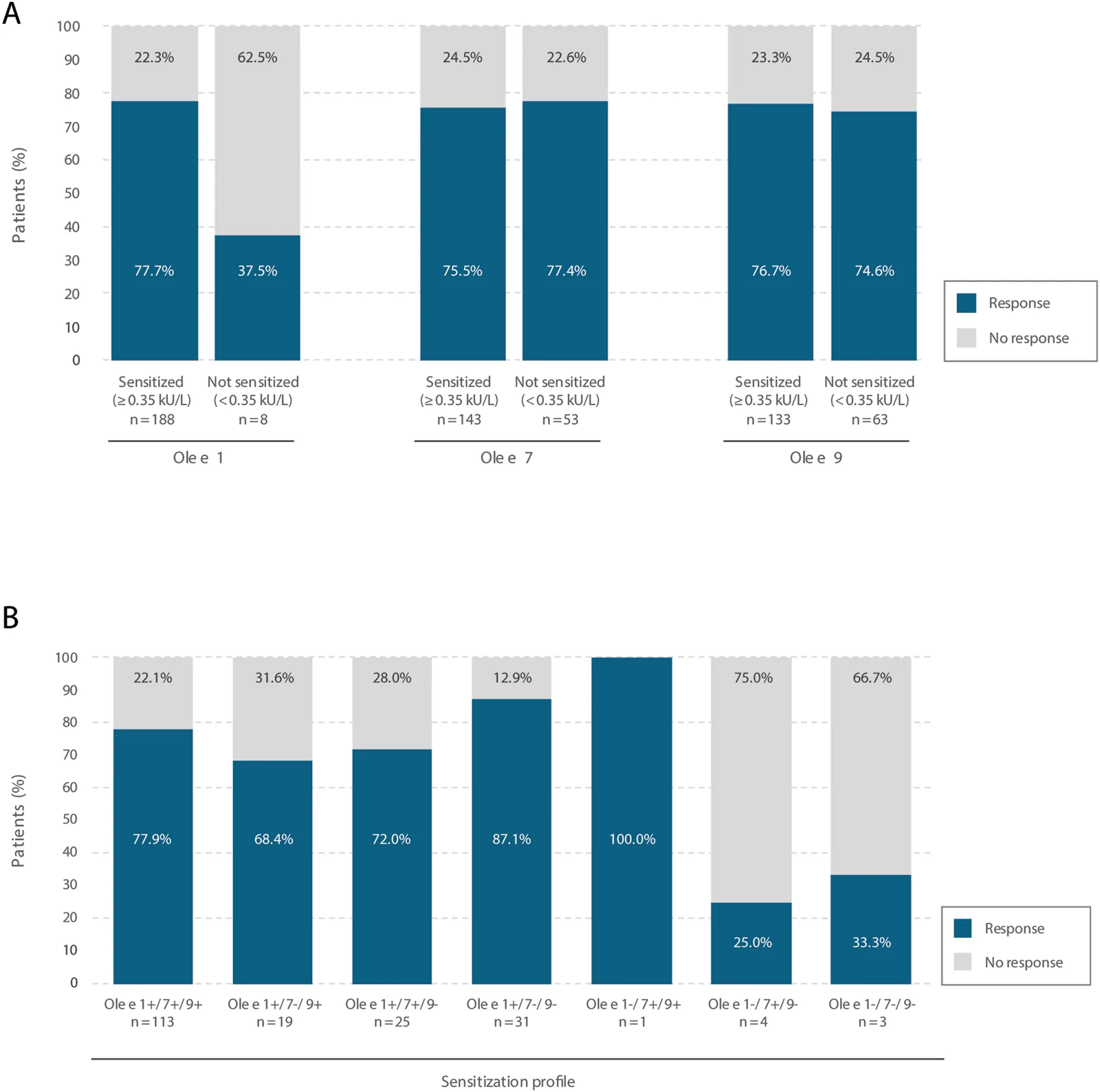
**Clinical response to allergen immunotherapy according to sensitization profile**. Percentage of patients showing a response (dark shading) or no response (light shading) to allergen immunotherapy stratified by their sensitization to olive pollen allergens. **(A)** Patients categorized according to sensitization to individual allergens (Ole e 1, Ole e 7, Ole e 9). **(B)** Patients categorized according to sensitization to multiple allergens, as indicated

### Reduction in emergency department visits and medication use

Between baseline and the one-year follow-up, the proportion of patients with at least 1 emergency department visit decreased from 33.0% (60/182) to 0% (0/182). Lower use of several medications was also observed at 1 year compared with baseline: oral corticosteroids decreased from 14.8% (27/182) to 3.8% (7/182), short-acting β_2_-agonists from 80.8% (147/182) to 46.7% (85/182), eye drops from 47.3% (86/182) to 31.9% (58/182), and nasal corticosteroids from 43.4% (79/182) to 33.5% (61/182). Antihistamine use showed a more modest decrease, from 97.3% (177/182) to 93.4% (170/182) (*p* < 0.001 for all comparisons) (Fig. 4A). In addition, 16 patients (8.8%) experienced a reduction in maintenance asthma medication during follow-up. Among these patients, 7 (43.8%) reduced treatment from 2 to 1 medication, 2 (12.5%) from 3 to 1, 5 (31.3%) from 3 to 2, 1 (6.3%) from 4 to 3, and 1 (6.3%) from 5 to 4 medications (data not shown). Similar reductions in emergency department visits and medication use were observed across sensitization subgroups defined by Ole e 1, Ole e 7, and Ole e 9 status (Fig. 4B).

**Fig. 4:**
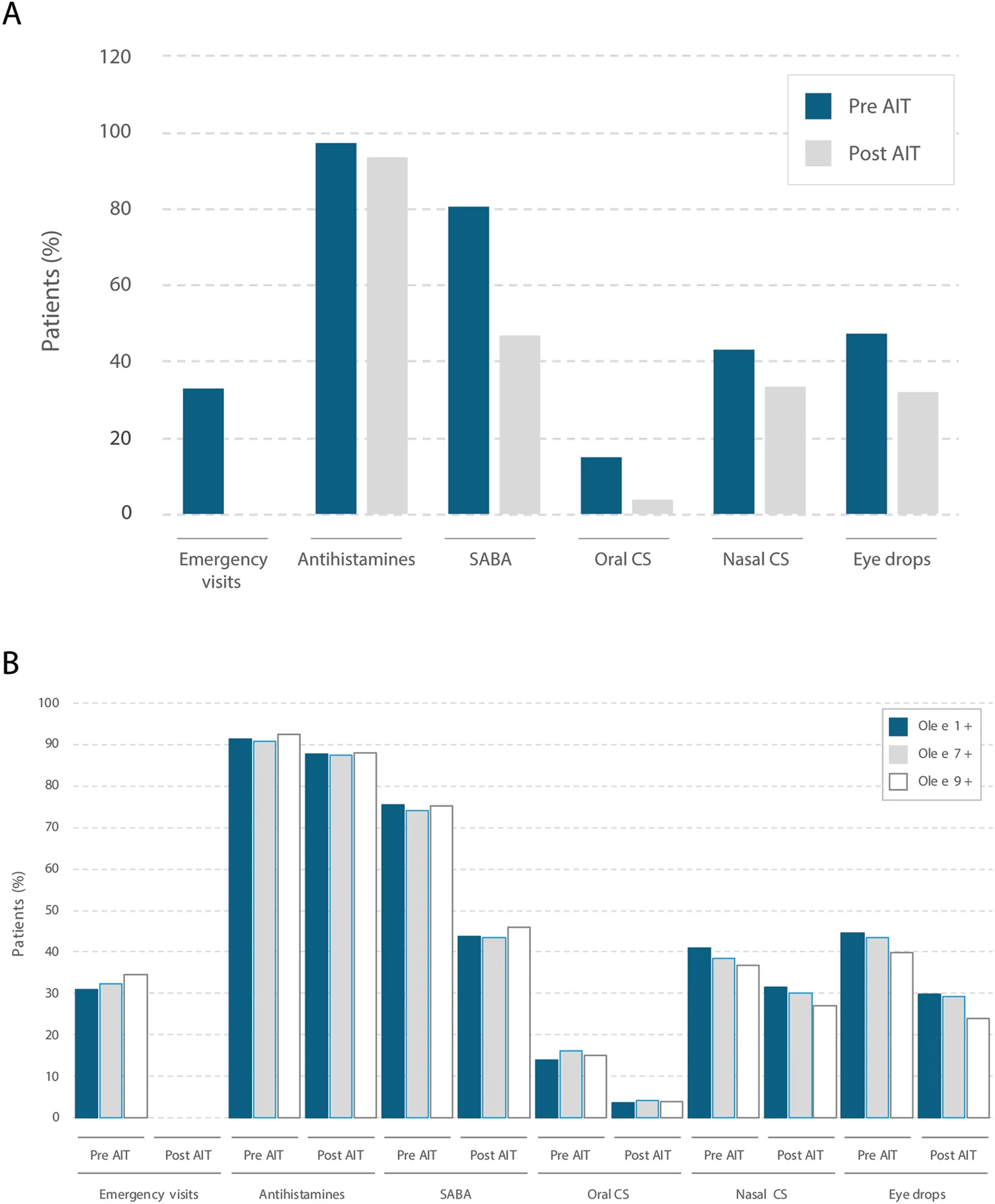
**Clinical benefit of allergen immunotherapy according to sensitization profile. (A)** Percentage of patients attending the emergency service and using the indicated medications before (Pre, dark bars) and after (Post, light bars) allergen immunotherapy (AIT), irrespective of sensitization status. (**B**) Percentage of patients attending the emergency service and using the indicated medications before (Pre) and after (Post) AIT, stratified by sensitization to individual olive pollen allergens: Ole e 1 (dark bars), Ole e 7 (light bars), and Ole e 9 (white bars)

At the one-year follow-up, reductions in maintenance asthma medication were observed in 8.5% of Ole e 1-sensitized patients, 8.4% of Ole e 7-sensitized patients, and 9.8% of Ole e 9-sensitized patients. Overall, Ole e 1-negative patients required less baseline treatment than Ole e 1-positive patients, resulting in a smaller reductions in the use of intranasal corticosteroids, eye drops, or other maintenance therapies during follow-up. In contrast, clinical outcomes in Ole e 7– and Ole e 9–negative patients were generally comparable to those observed in sensitized patients. Reductions in maintenance asthma medication use among non-sensitized patients were 0.0% for Ole e 1, 30.2% for Ole e 7, and 4.8% for Ole e 9 (data not shown).

### Association between tolerance to immunotherapy and clinical response

Among the 189 patients who tolerated AIT, 147 (77.8%) met the clinical response criteria, compared with 2 of 7 patients who experienced an adverse reaction (28.6%). This association was statistically significant (χ^2^, *p* = 0.0028). Similar trends were observed across sensitization subgroups. Among Ole e 1-sensitized patients, 79.6% of patients without adverse reactions (144/181) met the response criteria versus 28.6% (2/7) of those with adverse reactions (*p* = 0.0015); for Ole e 7, the corresponding proportions were 77.4% (106/137) versus 33.3% (2/6) (*p* = 0.0141); and for Ole e 9, 78.1% (100/128) versus 40.0% (2/5) (*p* = 0.0479) (Fig. 5).

**Fig. 5:**
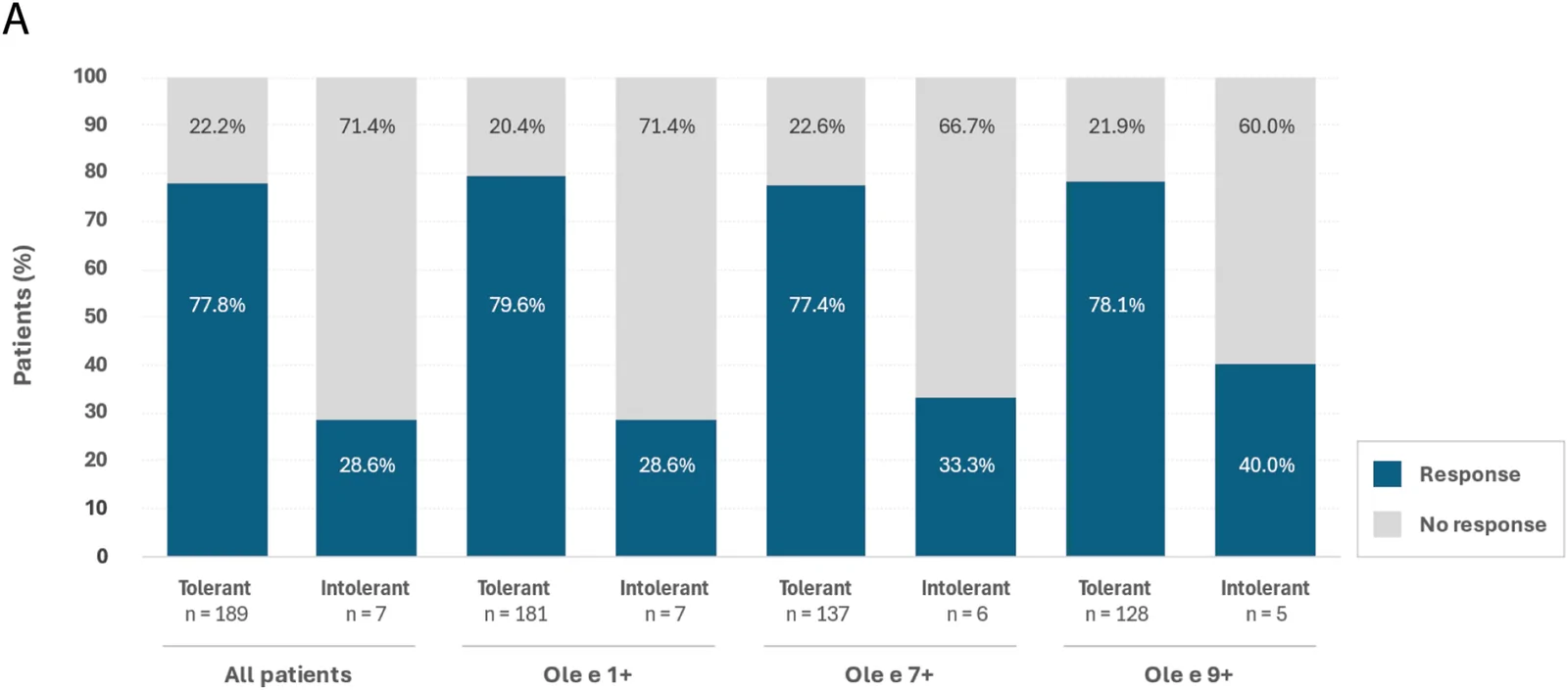
Percentage of patients showing a response (dark shading) or no response (light shading) to allergen immunotherapy according to their treatment tolerance status, as indicated

### Association between patient characteristics and incidence of adverse reactions

No significant associations were found between the incidence of adverse reactions and the following variables: age (*p* = 0.723), sex (*p* = 0.869), family history of olive allergy (*p* = 0.391), presence of atopic dermatitis (*p* = 0.201), presence of food allergy (*p* = 0.612), or duration of disease (*p* = 0.657). There was also no association with the molecular sensitization profile, either in terms of total IgE (*p* = 0.364), specific IgE to the whole olive allergen extract (*p* = 0.089), specific IgE to individual allergenic components (Ole e 1, *p* = 0.144; Ole e 7, *p* = 0.439; Ole e 9, *p* = 0.504), positive/negative presence of IgE to single allergenic components (± Ole e 1, *p* = 0.578; ± Ole e 7, *p* = 0.439; ± Ole e 9, *p* = 0.837), relative intensity of sensitization to single allergenic components (Ole e 1, *p* = 0.153; Ole e 7, *p* = 0.362; Ole e 9, *p* = 0.929), multiple sensitization profiles (Ole e 1^+^/Ole e 7^+^/Ole e 9^+^, *p* = 0.978; Ole e 1^+^/Ole e 7^−^/Ole e 9^+^, *p* = 0.676; Ole e 1^+^/Ole e 7^+^/Ole e 9^−^, *p* = 0.201; Ole e 1^+^/Ole e 7^−^/Ole e 9^−^, *p* = 0.243; Ole e 1^−^/Ole e 7^+^/Ole e 9^+^, *p* = 0.847) or the number of positive allergenic components (ie, 0, 1, 2, 3) (*p* = 0.858).

## Discussion

This retrospective observational study of 196 patients with ARD due to olive pollen allergy in Jaén, Spain, provides real-world data from a region characterized by exceptionally high environmental exposure. In this real-world cohort, SCIT demonstrated a favorable safety profile across a broad spectrum of olive pollen sensitization patterns, and was associated with clinically meaningful clinical improvements after 1 year of follow-up. Adverse reactions were uncommon and consisted predominantly of mild, delayed local reactions, while most patients met the predefined clinical response criteria, including documented reductions in emergency department visits and in the use of both maintenance and rescue medications. However, given the retrospective and uncontrolled nature of the study, these findings should be interpreted as associations observed during follow-up rather than effects attributed exclusively to SCIT.

Chronic exposure to high allergenic loads has been shown to influence sensitization patterns and immune responses. In regions with low-to-moderate olive pollen exposure, sensitization to the major allergen Ole e 1 stabilizes at approximately 80%, whereas both the prevalence and specific IgE levels of minor allergens such as Ole e 7 and Ole e 9 increase progressively along the exposure gradient, becoming highly prevalent in areas with extremely high pollen counts.^17^^,^^31^ Our results are consistent with this environmental gradient effect, with sensitization rates of 73.0% for Ole e 7 and 67.9% for Ole e 9, and mean sIgE levels exceeding those previously reported both in regions with lower exposure and in earlier studies conducted in Jaén itself.^18^^,^^32^^,^^33^

Previous studies have suggested that in areas of intense olive pollen exposure, Ole e 7 may act as a primary sensitizer independent of Ole e 1, with a substantial proportion of Ole e 1–negative patients showing reactivity to Ole e 7 ^18^ In our cohort, 62.5% of Ole e 1–negative patients were sensitized to Ole e 7, and up to half were monosensitized to this allergen, supporting its clinical relevance in this population.^16^

Sensitization to Ole e 7 and Ole e 9 has been proposed as a marker of panallergen reactivity rather than genuine olive pollen sensitization, raising concerns about reduced tolerance to allergen immunotherapy and an increased risk of severe reactions. Consequently, it has been suggested that the clinical benefits of olive pollen immunotherapy may be largely confined to patients sensitized to Ole e 1 ^24^^,^^32^ However, our findings challenge this assumption. In a cohort of nearly 200 patients living in a region with intense olive pollen exposure, SCIT was well tolerated across sensitization profiles, with only 7 treatment-related adverse reactions reported, most of which were mild delayed local reactions. The only systemic reaction occurred during the build-up phase in a child with well-controlled asthma who was receiving a mixed olive/grass allergen preparation. The reaction developed after the first dose, which was administered outside the olive pollen season, and resolved rapidly after treatment with inhaled salbutamol and systemic corticosteroids. Immunotherapy was continued without interruption, and no further local or systemic adverse reactions occurred during the remainder of the immunotherapy course. Although no conclusions can be drawn from a single case, the favorable outcome is consistent with the overall tolerability observed in this cohort. In addition, most patients met the predefined clinical response criteria, and no emergency department visits were recorded at the one-year follow-up. Nevertheless, given the absence of a control group, these improvements cannot be attributed with certainty to SCIT.

These favorable clinical outcomes were also observed among patients highly sensitized to Ole e 7, a molecular component historically associated with more severe allergic phenotypes.^17^^,^^34^ In this regard, a recent large study conducted in Jaén including 1111 olive pollen–allergic patients, encompassing the present cohort, reported that sensitization to Ole e 9 was associated with increased asthma severity, whereas sensitization to Ole e 7 was associated with a higher prevalence, but not greater severity, of asthma.^31^ Although the number of Ole e 1–negative patients in our study was limited, it is noteworthy that more than one-third achieved the predefined clinical response criteria at the one-year follow-up.

From a clinical perspective, complete allergen extracts may offer broad coverage of relevant allergenic components irrespective of individual sensitization profiles. Proteomic analyses have shown that depigmented polymerized olive pollen extracts contain both major and minor allergens, including Ole e 1, Ole e 7, and Ole e 9 ^34^ This comprehensive allergen representation may have contributed to the favorable outcomes observed across different molecular sensitization profiles; however, whether extract composition influenced these outcomes remains uncertain and warrants further investigation.

No significant association was observed between sensitization profile and the occurrence of adverse reactions. Within the limitations of the sample size and retrospective design, sensitization to Ole e 7 or Ole e 9 was not associated with poorer SCIT tolerance in this cohort.

This study has several strengths and limitations. The main strength is the integration of component-resolved diagnostics with real-world clinical outcomes in a population exposed to exceptionally high olive pollen levels. One limitation is that clinical response was not assessed using standardized pulmonary function tests, such as spirometry or PEF. Owing to the retrospective real-world design, these measurements were not consistently available at predefined time points for all patients and therefore could not be included in the analysis. Instead, clinical response was defined by patient-reported improvement confirmed by the treating physician and supported by improvement in at least 1 documented clinical or healthcare-utilization outcome, including rescue or maintenance medication use, systemic corticosteroid use, or emergency department attendance. Adverse reactions were assessed separately as a safety and tolerability outcome and were not included in the clinical response definition. Another limitation is that the study was not designed to distinguish between severe asthma and difficult-to-treat asthma according to current GINA definitions. Asthma severity was classified using the recommendations routinely applied during the study period, and only a small proportion of patients had severe persistent asthma. Consequently, our findings may not be generalizable to patients with severe or difficult-to-treat asthma. In addition, the absence of a control group limits the interpretation of the changes observed between baseline and 1 year of follow-up. Individual differences in environmental pollen exposure, management of concomitant conditions, adherence to maintenance medication, regression to the mean, and other unmeasured clinical factors may have contributed to the observed outcomes. As these variables were not systematically recorded or controlled, the relative contribution of SCIT to the clinical improvements observed during follow-up cannot be established with certainty.

Future prospective controlled studies incorporating serial pulmonary function testing, validated asthma control questionnaires, and standardized assessment of potential confounding variables are needed to provide a more comprehensive evaluation of treatment effectiveness and to further validate our findings. Such studies may also help inform potential updates to current guidelines, such as those of the EAACI, which currently do not recommend allergen immunotherapy in patients sensitized to Ole e 7 ^35^

## Conclusion

In this retrospective cohort, SCIT with a depigmented polymerized olive pollen extract was well tolerated, and sensitization to Ole e 7 or Ole e 9 was not associated with an increased frequency of adverse reactions. Favorable clinical outcomes were observed at 1 year across molecular sensitization profiles. However, given the absence of a control group and the lack of systematic assessment of potential confounding factors, the extent to which these improvements were attributable to SCIT remains uncertain. Prospective controlled studies are warranted to confirm these findings and to further optimize immunotherapy strategies for olive pollen-allergic patients in regions with high environmental exposure.

## Authorships

MAV, ALG, MBB, CRG, and PdMT made substantial contributions to the conception or design of the work, as well as to data acquisition and interpretation. GSF performed the data analysis and contributed to interpretation of data. All authors participated in drafting the manuscript and revising it critically for important intellectual content, approved the final version for publication, and agreed to be accountable for all aspects of the work, ensuring that questions related to the accuracy or integrity of any part of the study are appropriately investigated and resolved.

## Ethics approval

Patients' data were extracted from clinical records and collected in a case report form in an anonymized fashion. Patient informed consent was not required and was deemed unnecessary by the Research Ethics Committee reviewing the protocol. This study followed the General Data Protection Regulation (EU) 2016/679 (GDPR) and Organic Law March 2018 of December 5 on the Protection of Personal Data and guarantee of digital rights in Spain. The study was developed in accordance with ethical principles originating from the latest version of the Helsinki Declaration accepted by local authorities and in line with Good Clinical Practice (GCP) and the requirements of current Spanish regulations. This study was approved by the Research Ethics Committee of the Jaén province.

## Submission declaration

The authors confirm their manuscript is original, has not been published before, is not currently being considered for publication elsewhere. The preliminary results of this study were included in a poster presented at the 35th Congress of the Spanish Society of Allergy and Clinical Immunology (SEAIC), held in Granada, Spain, October 1–4, 2025 (Poster #157).

Authors consent for publication in WAO Journal.

## Declaration of Generative AI and AI-assisted technologies in the writing process

An AI-based language assistance tool was used to revise the manuscript for grammar, style, and readability. The tool did not contribute to the study design, data analysis, interpretation, or scientific conclusions. All output was reviewed, edited where necessary, and approved by the authors.

## Funding

This study did not receive funding.

The data that support the findings of this study are available from the corresponding author upon reasonable request.

## Conflict of interest

MAV has received speaker honoraria from Allergy Therapeutics, Inmunotek, ASAC Pharmaceutical Immunology, Roxall, LETI Pharma, GlaxoSmithKline, AstraZeneca, Chiesi, and Sanofi. GSF is a full-time employee of LETI Pharma. ALG, MBB, CRG and PdMT declare no conflicts of interest. The authors report no competing interests.
